# Missense mutations of *MLH1* and *MSH2* genes detected in patients with gastrointestinal cancer are associated with exonic splicing enhancers and silencers

**DOI:** 10.3892/ol.2013.1243

**Published:** 2013-03-11

**Authors:** MING ZHU, HUI-MEI CHEN, YA-PING WANG

**Affiliations:** 1 Jiangsu Key Laboratory of Molecular Medicine, Nanjing University School of Medicine, Nanjing 210093;; 2 Department of Molecular Biology, Jiangsu Institute of Cancer Research, Nanjing 210009, P.R. China

**Keywords:** gastrointestinal cancer, missense mutation, exonic splicing enhancer, exonic splicing silencer, *MSH2*, *MLH1*

## Abstract

The *MLH1* and *MSH2* genes in DNA mismatch repair are important in the pathogenesis of gastrointestinal cancer. Recent studies of normal and alternative splicing suggest that the deleterious effects of missense mutations may in fact be splicing-related when they are located in exonic splicing enhancers (ESEs) or exonic splicing silencers (ESSs). In this study, we used ESE-finder and FAS-ESS software to analyze the potential ESE/ESS motifs of the 114 missense mutations detected in the two genes in East Asian gastrointestinal cancer patients. In addition, we used the SIFT tool to functionally analyze these mutations. The amount of the ESE losses (68) was 51.1% higher than the ESE gains (45) of all the mutations. However, the amount of the ESS gains (27) was 107.7% higher than the ESS losses (13). In total, 56 (49.1%) mutations possessed a potential exonic splicing regulator (ESR) error. Eighty-one mutations (71.1%) were predicted to be deleterious with a lower tolerance index as detected by the Sorting Intolerant from Tolerant (SIFT) tool. Among these, 38 (33.3%) mutations were predicted to be functionally deleterious and possess one potential ESR error, while 18 (15.8%) mutations were predicted to be functionally deleterious and exhibit two potential ESR errors. These may be more likely to affect exon splicing. Our results indicated that there is a strong correlation between missense mutations in *MLH1* and *MSH2* genes detected in East Asian gastrointestinal cancer patients and ESR motifs. In order to correctly understand the molecular nature of mutations, splicing patterns should be compared between wild-type and mutant samples.

## Introduction

The incidence and mortality rates of gastric cancer and colorectal cancer are among the highest among malignant tumors in East Asia. Germline mutations of mismatch repair (MMR) genes are responsible for the majority of hereditary nonpolyposis colorectal cancer (HNPCC) cases. The MMR genes *MSH2* (OMIM No. 609309) and *MLH1* (OMIM No. 120436) are considered to be the two major genes implicated in HNPCC (1,2). Carriers of germline mutations of *MSH2* and *MLH1* genes have a 4-fold greater risk of gastric cancer compared with normal individuals, as well as a high risk of colorectal cancer. These two genes are associated with gastrointestinal cancer susceptibility.

Missense mutations are among the most common types of mutations underlying inherited human diseases. The deleterious effects of missense mutations are usually attributed to their effects on the structure or function of a protein. The assumption may be misleading, as the mutations that affect the sequences that are important for splicing modulation are likely to have a profound effect on the translated product. It has become increasingly clear that exonic point mutations located outside the splice sites may affect pre-mRNA splicing and thereby cause disease (3,4). Correct pre-mRNA splicing not only requires that the splice site sequences are present at the exon-intron borders, but is also critically dependent on additional intronic and exonic regulatory sequences (5). Those present in exons and with the capacity of enhancing splicing are called exonic splicing enhancers (ESEs) and those with the capacity of inhibiting the splicing are the exonic splicing silencers (ESSs). Generally, these classes of elements are called exonic splicing regulators (ESRs). Consequently, mutations located in ESE or ESS elements may affect splicing. The significance and prevalence of this phenomenon may have been significantly underestimated, as the majority of studies of disease-related genes are limited to the analysis of genomic DNA.

The majority of enhancer sequences within exons have been found to bind members of the serine/arginine-rich (SR) protein family, while many silencing elements are bound by members of the heterogeneous nuclear ribonuclearprotein (hnRNP) family (6). ESEs are discrete, degenerate motifs of 6–8 nts located inside exons (7,8). The study of normal splicing suggests that the majority of exons contain at least one functional ESE site (7,9). ESE-bound SR proteins promote exon definition by directly recruiting and stabilizing the splicing machinery through protein-protein interactions (10), and/or by antagonizing the function of nearby silencer elements (11). The cores of ESSs are considered to be relatively short (6–10 nts). ESS-bound hnRNPs are proposed to mediate silencing through direct antagonism of the splicing machinery or by direct competition for overlapping enhancer binding sites. The intrinsic strength by which the splice sites are recognized by the spliceosome, as well as the antagonistic dynamics of proteins binding ESEs and ESSs, control a large proportion of exon recognition and alternative splicing. Therefore, exonic splicing regulatory sequences are now increasingly recognized as a major target and a common mechanism for disease-causing mutations leading to exon skipping in functionally diverse genes.

In this study, we used ESE-finder (12,13) and FAS-ESS (14) software to analyze the missense mutations of *MSH2* and *MLH1* genes detected in East Asian gastrointestinal cancer patients, and to assess whether these mutations hit the predicted ESE/ESS motifs and affected gene splicing.

## Materials and methods

### Subjects

A total of 114 missense mutations, 52 of *MSH2* and 62 of *MLH1*, detected in the gastrointestinal cancer patients, were serially collected for this study from published East Asian literature (15–44) (Table I). The majority of the investigated mutations were exclusively reported in East Asia (China, Japan and Korea), and some of the mutations were detected in different ethnicities. The study was approved by the Ethics Committee of Nanjing University, Nanjing, China.

### Potential ESE motif analysis

To identify the ESE motifs that were recognized by individual SR proteins, a PCR-based systematic evolution of ligands by exponential enrichment (SELEX) was used. During this approach, a natural splicing enhancer in a minigene was replaced by short, random sequences derived from an oligonucleotide library. The generated pool of minigenes was transfected into cultured cells, and spliced mRNAs were amplified by RT-PCR and sequenced (7). On the basis of the frequencies of the individual nucleotides at each position, a score matrix for each nucleotide in each position was calculated. This score matrix may be used to predict SR protein-specific ESEs (ESE-finder) (12,13).

We analyzed wild-type or mutant exon sequences from *MLH1* and *MSH2* genes in Table I with ESE-finder software using SR protein score matrices and threshold values, essentially as described previously (ESE-finder: http://rulai.cshl.edu/tools/ESE/) (12). Sequence motifs for the same or different SR proteins may overlap. We considered only the wild type or mutant sequence motifs with scores greater than or equal to the value of the threshold for the corresponding SR protein. The threshold values were as follows: SF2/ASF (IgM-BRCA1) heptamer motif, 1.867; SC35 octamer motif, 2.383; SRp40 heptamer motif, 2.670 and SRp55 hexamer motif, 2.676.

### Potential ESS motif analysis

To systematically identify ESS motifs, an *in vivo* splicing reporter system was developed to screen a library of random decanucleotides. The resulting library was transfected into cultured human 293 cells, and stably transfected cells were combined and sorted for GFP-expressing cells by fluorescence activated cell sorting (FACS) analysis. The fluorescence-activated screen for exonic splicing silencers (FAS-ESS, or FAS for short) yielded 176 ESS hexamers (FAS-hex2 set) (14).

We analyzed wild-type or mutant exon sequences from *MLH1* and *MSH2* genes in Table I with FAS-ESS software using a FAS-hex2 set (176 ESS hexamers), essentially as described previously (FAS-ESS: http://genes.mit.edu/fas-ess/) (14).

### SIFT analysis

Sorting Intolerant from Tolerant (SIFT) tool (accessible at http://sift.jcvi.org/) was applied to detect deleterious missense mutations (45,46). SIFT compiles a dataset of functionally linked protein sequences by searching the protein database using a PSI-BLAST algorithm. Subsequently, it builds an alignment from the homologous sequences with the query sequence and scans all positions in the alignment, as well as calculating the probabilities for amino acids at that position. The substitution at each position with normalized probabilities of a tolerance index or SIFT score of <0.05 are predicted to be deleterious or intolerant, while those ≥0.05 are predicted to be tolerant (45). In this study, reference sequence (RefSeq) ID or GenInfo Identifier (GI) number and substitutions were provided as inputs to the SIFT blink program (46). A total of 52 missense mutations in the *MSH2* gene (GI: 4557761) and 62 in the *MLH1* gene (GI, 463989) were analyzed for identification of deleterious variants.

## Results

### Potential ESE/ESS analysis of the mutations in MLH1 and MSH2 genes

**Figure 1:**
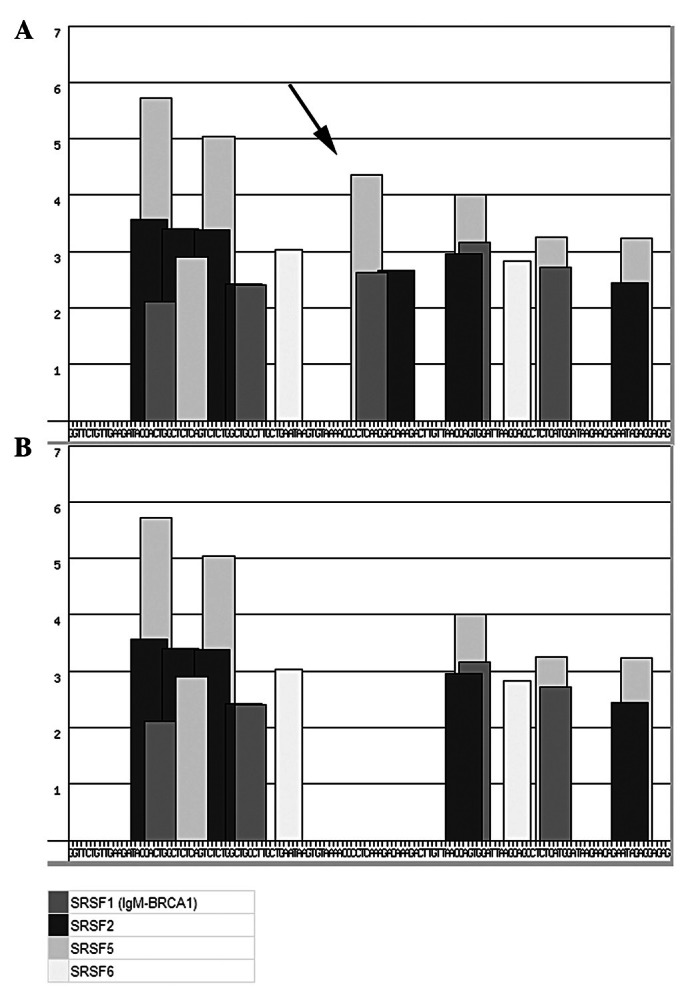
High-score splicing regulator (SR) protein motif analysis in *MSH2* exon 6 (A) and a single point mutation variant c.1012G>A (B). High-score motifs are shown in dark gray for SF2/ASF (IgM-BRCA1), black for SC35, light gray for SRp40 and white for SRp55, and only the scores above the threshold for each SR protein are shown. The height of each bar indicates the score value, and its width and placement on the x-axis represent the length of the motif (6–8 nt) and its position along the sequence. The arrow indicated that the c.1012G>A transversion in *MSH2* exon 6 affected a SF2/ASF motif, reducing the score from 2.620 to 0.840; a SC35 motif, reducing the score from 2.669 to 0.917; and a SRp40 motif, reducing the score from 4.353 to 1.971.

We analyzed wild-type or mutant exon sequences from *MLH1* and *MSH2* genes in Table I using SR protein score matrices and threshold values, essentially as described. Potential ESE motifs found in the mutations in the two genes are listed in Table I (Fig. 1). Some of the mutations may load in different potential ESE motifs. Of the 114 mutations analyzed, 47 (41.2%) mutations resulted in 68 ESE motif scores (24 SF2/ASF [IgM-BRCA1], 13 SC35, 19 SRp40 and 12 SRp55) being eliminated. However, 38 (33.3%) mutations created 45 ESE motif scores (12 SF2/ASF [IgM-BRCA1], 8 SC35, 14 SRp40 and 11 SRp55).

**Figure 2:**
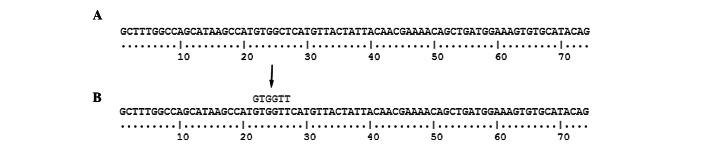
Fluorescence-activated screen for exonic splicing silencers (FAS-ESS) analysis of *MLH1* exon 4 (A) and a single point mutation variant c.332C>T (B). The arrow indicates that the c.332C>T transversion in *MLH1* exon 4 created a new ESS motif.

We analyzed wild-type or mutant exon sequences from *MLH1* and *MSH2* genes in Table I with FAS-ESS using the FAS-hex2 set (176 ESS hexamers), essentially as described previously. Potential ESS motifs found in the mutations in the two genes are listed in Table I (Fig. 2). Of the 114 mutations assessed, 9 (7.9%) mutations resulted in 13 ESS motif scores being eliminated. However, 17 (14.9%) mutations created 27 ESS motif scores.

Eliminating the potential ESE motif and creating the potential ESS motif have the same effect on exon exclusion. We named these mutations as potential ESR error mutations. In total, 56 (49.1%) mutations possessed a potential ESR error (Table II).

### Deleterious missense mutations predicted by the SIFT server

Eighty-one missense mutations (71.1%) were predicted to be deleterious with a tolerance index <0.05; the lower the tolerance score, the greater the functional consequence an amino acid residue substitution is expected to have (Table I). Additionally, 32 (28.1%) mutations were predicted to be deleterious with potential ESE motif losses, while 13 (11.4%) mutations were predicted to be deleterious with potential ESS motif gains. In total, 38 (33.3%) mutations were predicted to be deleterious with potential ESR errors (Table II).

## Discussion

The incidence and mortality rates of gastrointestinal cancer are among the highest malignant tumors in East Asia. MMR genes *MLH1* and *MSH2* have been known to play an important role in the pathogenesis of gastrointestinal cancer. At present, the majority of databases contain annotation data that are primarily or exclusively derived from genomic DNA analysis, and the effect of a mutation on the mRNA or on the encoded protein is usually predicted from the primary sequence, rather than by experimentally determining the mRNA expression and splicing patterns. Therefore, the majority of reported disease-associated alleles of these genes are small insertions, deletions or splice-site mutations that result in protein truncation. Thus, only a small number of amino acid substitutions in either gene have been described as deleterious missense mutations, yet a very large number of different unclassified variant alleles are routinely encountered in clinical and research laboratories. It is therefore necessary to functionally define these unclassified variants as deleterious alleles, low-penetrance alleles or benign polymorphisms.

In this study, we selected 114 missense mutations of *MSH2* and *MLH1* genes detected from East Asian gastrointestinal cancer patients in published studies. The ethnic group included Chinese, Japanese and Korean individuals. The missense mutations contribute to certain forms of cancer susceptibility in East Asian populations, but it was unclear whether these were the definite pathogenic mutations in gastrointestinal cancer.

The consequences of splicing unclassified variants found in the *MLH1* or the *MSH2* genes may be studied directly at the patient RNA level. However, the number of variants that may be tested for splicing alterations using patient RNA is limited by the difficulty of obtaining blood samples suitable for RNA extraction. The bioinformatic tools, the ESE-finder and FAS-ESS, may enable prediction of the splicing defect of the mutations. These tools have already been used successfully to predict ESEs/ESSs and their disruption in a variety of genes, including *ACF, BRCA1, BRCA2, FBN1, IGF1, PDHA1, SMN1, SMN2, TNFRSF5, CFTR, MlH1, MSH2, Tp53, MCAD* and others (3,7,47–52). Auclair *et al* conducted a systematic RNA screening of a series of 60 western patients who carried unrelated exonic or intronic mutations in the *MLH1* or *MSH2* genes (53). In addition, it was found that the potential correlation between aberrant splicing and prediction of ESE by the ESE-finder demonstrated a sensitivity of 80% and a specificity of 42%.

Under the conditions of the null hypothesis, there is no correlation between ESEs and mutations; the amount of ESE motif scores eliminated or created should be equal. However, in the present study, the amount of ESE losses (68) was 51.1% higher than ESE gains (45). This suggested that the mutations loaded in the potential ESE motifs were more likely to eliminate the ESE motif score, and that they affected gene splicing. Additionally, under the conditions of the null hypothesis, there is no correlation between ESSs and mutations; the amount of ESS motif scores eliminated or created should be equal. Conversely, in the present study, the amount of ESS gains (27) was 107.7% higher than the amount of ESS losses (13). This suggested that the mutations were more likely to create the ESS motif score and that they affected gene splicing, indicating that there is a strong association between missense mutations in *MLH1* and *MSH2* genes and ESE/ESS motifs. Some of the mutations should be splicing-related deleterious alleles.

As an upper limit for the estimate of the proportion of ESR-related mutations, we suggest that 56 (49.1%) mutations, which have lost ESE or gained ESS motifs, were deleterious for the reason that they disturbed functional splicing enhancers or or created functional splicing silencers, respectively. This approach was likely to overestimate the proportion of ESR-related pathogenic mutations. This is due to the fact that not all ESR motifs are true functional ESRs, and not all nucleotide substitutions in functional ESRs disturb their function.

According to previous studies, no extensive functional analysis was available for these mutations. We used the SIFT tool to functionally analyze the missense mutations. SIFT is a program that predicts the effect of amino acid substitutions on protein function, on the basis of sequence conservation during evolution and the nature of the amino acids substituted in a gene of interest. In total, 81 missense mutations (71.1%) were predicted to be deleterious with a tolerance index <0.05. Among them, 38 (33.3%) mutations were predicted to be deleterious and have at least one potential ESR error. Some of these may be pathogenic with exon exclusion.

Eliminating and creating the potential ESE motif has the same effect on exon exclusion. One mutation may eliminate one or more potential ESE motifs. The greater the number of potential ESE motifs eliminated, the more likely the mutation was to affect the ESE motifs. However, one mutation may create one or more potential ESS motifs. The greater the number of potential ESS motifs created, the more likely the mutation was to affect the ESS motifs. In total, 25 (21.9%) mutations eliminated at least two potential ESE motifs, or created at least two potential ESS motifs, or eliminated one or more potential ESE motifs and created one or more potential ESS motifs. These may be more likely to affect exon splicing. Among these, 18 (15.8%) mutations, c.1012G>A, c.1168C>T, c.1799C>T, c.1808A>G, c.2047G>A, c.2064G>A, c.2108C>A, c.2128G>T, c.2141C>T in *MSH2*; c.242C>T, c.318C>G, c.327T>G, c.332C>T, c.1186T>A, c.1561C>A, c.1907T>C, c.2059C>T, c.2263A>G in *MLH1,* were predicted to be deleterious in the SIFT analysis. These were the mutations that most likely affected exon splicing, and were denoted as ESR-relevant mutations. We proposed that some of these disrupted functional ESEs or created functional ESSs, leading to the creation of a misspliced message predicted to encode a truncated, non-functional protein. However, these data did not allow us to determine which of the SR protein/hnRNPs motifs were functional. Although it is unlikely that each motif was able to be recognized simultaneously, due to the overlap between them, it is possible that each motif was important in a different cell type, depending on the relative expression levels of SR protein/hnRNPs.

Several putative ESR sequences have been found in exons where they have been sought systematically, raising the possibility of functional redundancy. This may diminish the potential exon-skipping effect of a mutation in any one ESR. However, in cases where 3–10 putative ESE sequences occur within a single exon, a single ESE-disrupting base substitution may lead to efficient exon skipping. Fackenthal *et al* found that *BRCA2* T2722R was a deleterious allele that caused *BRCA2* exon 18 skipping (48), and Pagenstecher *et al* found a silent mutation *MLH1* c.1731G>A caused *MLH1* exon 15 skipping (54). However, a single ESS-creating base substitution may lead to efficient exon skipping. Oliveira *et al* found that POMGNT1 c.636C>T created a new ESS and caused *POMGNT1* exon 7 skipping (55), and Raponi *et al* found that *BRCA1* c.231G>T created a new ESS and caused *BRCA1* exon 6 skipping (56). This suggests that, at least in certain cases, individual ESRs may be critical for splicing even when other ESRs are present in the same exon. However, the splice mutations of *MLH1* and *MSH2* have been underestimated. The strong correlation between missense mutations with splicing enhancer/silencer motifs found in this study also suggested that splicing-related mutations in the two genes may be relatively common. The computer predictions do not always correlate with *in vivo* splicing defects. The predictable ESR error mutations require experimental analysis for validation in a further study.

In conclusion, our results indicated that there is a strong correlation between missense mutations in *MLH1* and *MSH2* genes detected in East Asian gastrointestinal cancer patients and ESR motifs. In total, 38 (33.3%) mutations were predicted to be functionally deleterious and possess one potential ESR error, while 18 (15.8%) mutations were predicted to be functionally deleterious with two potential ESR errors. These may be more likely to affect exon splicing. To truly understand the molecular nature of mutations, splicing patterns should be compared between wild-type and mutant samples.

## Figures and Tables

**Table I:** Pathogenic missense mutations analyzed in the splicing assay.

				ESE finder^a^							
Gene	Exon	Nucleotide change	Amino acid change	SF2/ASF	SC35	SRp40	SRp55	FAS ESSb	SIFT prediction	Ethnicity	Reference
MSH2	1	c.14C>A	p.Pro5His	−1/3					Damaging	Chinese	15
MSH2	1	c.23C>T	p.Thr8Met	−1/1	=1		+1/0		Tolerated	Chinese; Japanese	16, 17
MSH2	1	c.49G>T	p.Val17Phe	=1			+1/0	+1/0	Tolerated	Korean	18
MSH2	2	c.329A>C	p.Lys110Thr				+1/0		Tolerated	Japanese	19
MSH2	3	c.380A>T	p.Asn127Ile						Damaging	Korean	20
MSH2	3	c.505A>G	p.Ile169Val		=1		=1	+1/0	Tolerated	Chinese	21
MSH2	3	c.518T>G	p.Leu173Arg					−1/1	Damaging	Chinese	21
MSH2	3	c.529G>A	p.Glu177Lys				=1		Damaging	Chinese	22
MSH2	3	c.595T>C	p.Cys199Arg						Damaging	Chinese	23
MSH2	3	c.619G>T	p.Ala207Ser	−1/1	=1	−1/2			Tolerated	Chinese	24
MSH2	5	c.884A>G	p.Asp295Gly		=1			+1/0	Damaging	Korean	25
MSH2	6	c.968C>A	p.Ser323Tyr		=1	−1/1			Tolerated	Korean	18
MSH2	6	c.1004C>T	p.Thr335Ile						Damaging	Korean	18
MSH2	6	c.1012G>A	p.Gly338Arg	−1/1	−1/1	−1/1			Damaging	Chinese	26
MSH2	7	c.1108G>A	p.Ala370Thr				=1		Tolerated	Chinese	27
MSH2	7	c.1145G>A	p.Arg382His				=1		Damaging	Chinese	28
MSH2	7	c.1168C>T	p.Leu390Phe	−1/1		−1/1			Damaging	Chinese	21
MSH2	7	c.1223A>G	p.Tyr408Cys		+1/0	=1			Damaging	Chinese	21
MSH2	7	c.1225C>A	p.Gln409Lys			+1,−1/1			Damaging	Chinese	28
MSH2	7	c.1255C>A	p.Gln419Lys			=1			Tolerated	Chinese	21
MSH2	7	c.1261C>A	p.Leu421Met	−1/1			+1/0		Damaging	Chinese	21
MSH2	10	c.1516G>T	p.Asp506Tyr		=1				Damaging	Korean	29
MSH2	10	c.1571G>T	p.Arg524Leu			+1/0	−1/1		Damaging	Chinese	30
MSH2	11	c.1688A>C	p.Tyr563Ser		=1			−1/1	Damaging	Chinese	31
MSH2	12	c.1799C>T	p.Ala600Val	−1/1				+2/0	Damaging	Japanese	32
MSH2	12	c.1807G>T	p.Asp603Tyr		−1/1				Damaging	Chinese	33
MSH2	12	c.1808A>G	p.Asp603Gly		−1/1			+1/0	Damaging	Chinese	34
MSH2	12	c.1886A>G	p.Gln629Arg	+1/0					Tolerated	Chinese; Korean	18, 35
MSH2	12	c.1916A>G	p.His639Arg	+1/0					Damaging	Japanese	19
MSH2	12	c.1917T>A	p.His639Gln		=1				Damaging	Chinese	30
MSH2	12	c.1939G>A	p.Glu647Lys						Damaging	Japanese	19
MSH2	12	c.1955C>A	p.Pro652His		−1/1		+1/0		Damaging	Chinese	30
MSH2	12	c.1966T>C	p.Tyr656His				−1/1		Tolerated	Japanese	19
MSH2	13	c.2036T>C	p.Ile679Thr			+1/0			Damaging	Japanese	19
MSH2	13	c.2047G>A	p.Gly683Arg	+1,−1/1		−1/1		+2/2	Damaging	Chinese	30
MSH2	13	c.2064G>A	p.Met688Ile	−1/1	−2/2	+1/0			Damaging	Korean	36
MSH2	13	c.2087C>T	p. Pro696Leu						Damaging	Chinese	27
MSH2	13	c.2089T>C	p.Cys697Arg	+1/0		+1/0	+1/0		Damaging	Korean	37
MSH2	13	c.2108C>A	p.Ser703Tyr		=1		−1/1	+1/0	Damaging	Chinese	22
MSH2	13	c.2128G>T	p.Ala710Ser	−1/1	−1/1				Damaging	Chinese	26
MSH2	13	c.2141C>T	p.Ala714Val		=1			+4/0	Damaging	Korean	18
MSH2	13	c.2168C>T	p.Ser723Phe			=1			Damaging	Japanese	32
MSH2	13	c.2185A>G	p.Met729Val	+1/0	=1			+1/0	Damaging	Japanese	19
MSH2	13	c.2195C>T	p.Thr732Ile						Damaging	Japanese	19
MSH2	14	c.2425G>A	p.Glu809Lys	−1/1		+1/0			Tolerated	Chinese	27
MSH2	15	c.2479G>A	p.Gly827Arg					−1/2	Damaging	Chinese	33
MSH2	15	c.2516A>G	p.His839Arg				=1		Tolerated	Chinese	16
MSH2	15	c.2533A>G	p.Lys845Glu						Damaging	Japanese	17
MSH2	15	c.2564A>C	p.Gln855Pro		+1/0		+2/0	+2, −1/1	Tolerated	Korean	25
MSH2	16	c.2649T>G	p.Ile883Met						Tolerated	Korean	38
MSH2	16	c.2651T>C	p.Ile884Thr	+1/0		+1/1			Damaging	Korean	25
MSH2	16	c.2792A>C	p.Lys931Thr			−1/1			Damaging	Chinese	15
MLH1	1	c.107T>A	p.Ile36Asn						Damaging	Chinese	30
MLH1	2	c.122AT>TA	p.Asp41Val			+1/0		+1/1	Damaging	Chinese	27
MLH1	2	c.137G>T	p.Ser46Ile	−1/1		=1			Tolerated	Chinese	22
MLH1	2	c.158A>C	p.Glu53Ala			+1/0			Tolerated	Japanese	19
MLH1	2	c.194G>A	p.Gly65Asp			+2,−1/1			Damaging	Chinese	15
MLH1	2	c.199G>A	p.Gly67Arg	=1	+1/0				Damaging	Chinese	16
MLH1	2	c.205A>G	p.Arg69Gly						Damaging	Japanese	19
MLH1	3	c.229T>C	p.Cys77Arg				+1/0		Damaging	Chinese	27
MLH1	3	c.242C>T	p.Thr81Ile		−1/2	−1/2			Damaging	Japanese	19
MLH1	3	c.250A>G	p.Lys84Glu		+1/0				Damaging	Chinese	27
MLH1	4	c.318C>G	p.Ser106Arg		−1/1		−1/1		Damaging	Korean	18
MLH1	4	c.327T>G	p.His109Gln				−1/1	+1/0	Damaging	Korean	18
MLH1	4	c.332C>T	p.Ala111Val	−1/1	=1		−1/1	+1/0	Damaging	Japanese	17
MLH1	4	c.350C>T	p.Thr117Met			=1			Damaging	Chinese	27
MLH1	6	c.498A>C	p.Leu166Phe						Tolerated	Chinese	30
MLH1	7	c.572G>T	p.Ser191Ile					−4/4	Damaging	Chinese	30
MLH1	8	c.637G>T	p.Val213Leu	−2/2		−1/1			Tolerated	Chinese	16
MLH1	8	c.649C>T	p.Arg217Cys	−1/1					Damaging	Japanese; Korean	29, 32
MLH1	8	c.655A>G	p.Ile219Val	=1			=1		Tolerated	Chinese; Japanese	17; 21
MLH1	8	c.677 G>A	p.Arg226Gln			−1/1			Damaging	Korean	25
MLH1	9	c.701A>G	p.Glu234Gly			−1/1	−1/1	+3/0	Tolerated	Korean	18
MLH1	9	c.790C>T	p.His264Tyr						Damaging	Chinese	33
MLH1	10	c.793C>T	p.Arg265Cys			=1			Damaging	Chinese	27
MLH1	10	c.845C>G	p.Ala282Gly	−1/1			=1		Tolerated	Korean	39
MLH1	11	c.908T>A	p.Val303Glu				=1		Damaging	Chinese	30
MLH1	11	c.949C>A	p.Leu317Met			−1/1		−1/1	Damaging	Chinese	30
MLH1	11	c.962G>T	p.Ser321Ile		−1/1		−1/1		Tolerated	Korean	18
MLH1	11	c.985C>A	p.His329Asn	+1/0			−1/2		Tolerated	Chinese	22
MLH1	11	c.1038G>T	p. Gln346His	=1					Damaging	Chinese	27
MLH1	12	c.1151T>A	p.Val384Asp		=1		−1/1	−2/2	Damaging	Chinese	40
MLH1	12	c.1178T>C	p.Leu393Pro						Damaging	Chinese	27
MLH1	12	c.1186T>A	p.Phe396Ile		−1/1	=1	−1/1		Damaging	Korean	25
MLH1	12	c.1198C>G	p.Leu400Val	+1,−1/1	=1	−1/1			Tolerated	Chinese	31
MLH1	12	c.1246A>G	p.Lys416Glu						Tolerated	Chinese	30
MLH1	13	c.1453G>C	p.Asp485His		=1				Tolerated	Korean	18
MLH1	14	c.1561C>A	p.Leu521Ile	−1/1		−1/1			Damaging	Japanese	19
MLH1	14	c.1576C>T	p.His526Tyr				=1		Damaging	Japanese	19
MLH1	14	c.1625A>T	p.Gln542Leu	=1		=1	−1/2		Damaging	Japanese; Korean	39, 41
MLH1	14	c.1646T>C	p.Leu549Pro						Damaging	Korean	29
MLH1	15	c.1681T>C	p.Tyr561His	+1/0		=1		+1, −1/1	Damaging	Chinese	22
MLH1	15	c.1721T>C	p.Leu574Pro	=1	+1/1	=1		+1/0	Damaging	Japanese; Korean	39, 41
MLH1	16	c.1742C>T	p.Pro581Leu	−2/2		+1/0			Tolerated	Chinese	42
MLH1	16	c.1744C>G	p.Leu582Val	=2	+1/0				Tolerated	Japanese	41
MLH1	16	c.1763T>C	p.Leu588Pro	+1/0					Damaging	Japanese	17
MLH1	16	c.1799A>G	p.Glu600Gly	=1					Damaging	Chinese	22
MLH1	16	c.1823C>A	p.Ala608Asp						Damaging	Chinese	30
MLH1	17	c.1900G>A	p.Gly634Arg						Damaging	Japanese	19
MLH1	17	c.1905C>G	p.Asn635Lys		−1/1				Tolerated	Korean	18
MLH1	17	c.1907T>C	p.Leu636Pro		−1/1	−1/1			Damaging	Korean	39
MLH1	17	c.1918C>T	p.Pro640Ser			−1/1			Damaging	Korean	39
MLH1	17	c.1942C>T	p.Pro648Ser	−1/1	+1/0		+1/0		Damaging	Chinese	22
MLH1	17	c.1984A>C	p.Thr662Pro	+1/0	=1	−1/1			Tolerated	Korean	38
MLH1	17	c.1988A>C	p.Glu663Ala			=1			Tolerated	Chinese	30
MLH1	18	c.2038T>C	p.Cys680Arg	+1/0					Damaging	Chinese	30
MLH1	18	c.2041G>A	p.Ala681Thr						Damaging	Chinese	43
MLH1	18	c.2042C>T	p.Ala681Val				+1/0		Damaging	Chinese	44
MLH1	18	c.2059C>T	p.Arg687Trp	−2/2					Damaging	Japanese	32
MLH1	18	c.2101C>A	p.Gln701Lys		=1	+1/0			Tolerated	Chinese	21
MLH1	19	c.2168C>A	p.Ala723Asp		+1/0				Damaging	Korean	39
MLH1	19	c.2170T>A	p.Leu724Met						Tolerated	Korean	39
MLH1	19	c.2210A>T	p.Asp737Val	=1				−1/1	Damaging	Japanese	19
MLH1	19	c.2263A>G	p.Arg755Gly			+1,−1/1		+3/0	Damaging	Chinese	27

a Number of ESE motifs added or abrogated from the mutation/number of ESE motifs in the normal allele.

b Number of ESS motifs added or abrogated from the mutation/number of ESS motifs in the normal allele.

**Table II:** Potential ESR errors detected in the mutations of the two genes.

	Total mutations	ESE eliminated	ESS created	ESR error	Two ESRs error
Total mutations	114 (100.0%)	47 (41.2%)	17 (14.9%)	56 (49.1%)	25 (21.9%)
SIFT deleterious	81 (71.1%)	32 (28.1%)	13 (11.4%)	38 (33.3%)	18 (15.8%)

ESR error; mutations that have lost one ESE motif or gained one ESS motif. Two ESRs error; mutations that have eliminated at least two potential ESE motifs, or created at least two potential ESS motifs, or eliminated one or more potential ESE motifs and created one or more potential ESS motifs. ESR, exonic splicing regulator; ESE, exonic splicing enhancer; ESS, exonic splicing silencer.
